# Contribution of Individual Polyphenols to Antioxidant Activity of *Cotoneaster bullatus* and *Cotoneaster zabelii* Leaves—Structural Relationships, Synergy Effects and Application for Quality Control

**DOI:** 10.3390/antiox9010069

**Published:** 2020-01-12

**Authors:** Agnieszka Kicel, Aleksandra Owczarek, Paulina Kapusta, Joanna Kolodziejczyk-Czepas, Monika A. Olszewska

**Affiliations:** 1 Department of Pharmacognosy, Faculty of Pharmacy, Medical University of Lodz, 1 Muszynskiego, 90-151 Lodz, Poland; aleksandra.owczarek@umed.lodz.pl (A.O.); monika.olszewska@umed.lodz.pl (M.A.O.); 2 Department of General Biochemistry, Faculty of Biology and Environmental Protection, University of Lodz, 141/143 Pomorska, 90-236 Lodz, Poland; paulinakapusta94@wp.pl (P.K.); joanna.kolodziejczyk@biol.uni.lodz.pl (J.K.-C.)

**Keywords:** *Cotoneaster*, polyphenols, isolation, standardization, antioxidants, peroxynitrite, human plasma, synergy, HPLC-PDA, validation

## Abstract

*Cotoneaster* plants are sources of traditional medicines and dietary products, with health benefits resulting from their phenolic contents and antioxidant activity. In this work, active markers of the leaves of *C. bullatus* and *C. zabelii* were characterized and evaluated in an integrated phytochemical and biological activity study. Based on UHPLC-PDA-ESI-MS^3^ analysis, twelve analytes were preselected from the constituents of the hydromethanolic leaf extracts, and two of them—caffeoylmalic acid and quercetin 3--*O*-β-d-(2″--*O*-β-d-xylopyranosyl)galactopyranoside (QPH)—were isolated for full identification (NMR spectroscopy: ^1^H, ^13^C, COSY, HMBC, HMQC). All selected phenolics contributed to the antioxidant activity of the extracts, which was demonstrated in chemical in vitro tests (DPPH, FRAP, and TBARS) and in a biological model of human plasma exposed to oxidative/nitrative stress induced by peroxynitrite. This contribution was partly due to the synergy between individual polyphenols, evidenced by an isobolographic analysis of the interactions of (–)-epicatechin, chlorogenic acid, and QPH as representatives of three classes of *Cotoneaster* polyphenols. All twelve markers, including also neochlorogenic acid, cryptochlorogenic acid, procyanidin B2, procyanidin C1, rutin, hyperoside, isoquercitrin, and quercitrin, were thus applied as calibration standards, and a fast, accurate, reproducible, and fully validated RP-HPLC-PDA method for quality control and standardization of the target extracts was proposed.

## 1. Introduction

The genus *Cotoneaster* Medikus (Rosaceae) comprises about 500 species native to central and southern China and naturalized in Europe, where they are extensively cultivated as ornamental plants. Various *Cotoneaster* representatives are used in traditional Asian medicine for their cardiotonic, hypotensive, diuretic, antispasmodic, expectorant, astringent and antiviral properties, and are indicated, e.g., for cardiac complaints, diabetes mellitus, nosebleeds, hematemesis and excessive menstruation [1,2,3]. Based on the previous studies, phenolics from the group of mono- and oligomeric flavan-3-ols, quinic acid pseudodepsides and flavonol glycosides may be responsible for these activities [2,4,5,6,7]. As plants rich in polyphenols—the main group of natural antioxidants—the *Cotoneaster* species have also been noticed as a source of extracts with potential application in oxidative stress-related pathologies [6,7]. In this context, among the species cultivated in Poland, *C. bullatus* and *C. zabelii* were characterized by the highest polyphenol content and extraction yield as well as the best antioxidant activity parameters [5,6,7]. In particular, the leaf extracts from both species exhibited relevant direct scavenging properties towards reactive oxygen/nitrogen species (ROS/RNS), protected the protein and lipid components of human plasma against ROS/RNS-related oxidative/nitrative damage, enhanced the non-enzymatic antioxidant capacity of plasma, and inhibited lipoxygenase—a pro-oxidant and pro-inflammatory enzyme [5,6]. On the other hand, although both species contain the same main groups of polyphenols (flavan-3-ols, caffeoyl acid derivatives, flavonoids), there are some quantitative and qualitative differences, e.g., some compounds are present only in one of the species [5,6].

The variable composition of plant extracts may be reflected in differences in their antioxidant efficacy and safety of usage, and thus is a major concern regarding their therapeutic applications. Therefore, for herbal products a large emphasis is put on quality control and standardization aspects, especially on the reliable identification and quantification of potentially active constituents [8,9]. To select a proper methodology for those tasks, and, in particular, to choose proper analytical (active) markers, it is important to know which constituent, and to what degree, is responsible for the investigated effects. In case of the active extracts from *Cotoneaster* leaves, only their overall activity has been investigated to date, and there is still missing information regarding the molecular structure of the main ingredients and the influence of particular constituents on their antioxidant activity. Moreover, as the target plants contain multiple groups of phenolics, some interactions between them may result in synergic or antagonistic effects influencing the total activity of the extracts. 

Therefore, the aim of the study was to evaluate the contribution of individual phenolics, as well as groups of phenolics, to the antioxidant activity of the leaf extracts of *C. bullatus* and *C. zabelii*. Based on previous UHPLC-MS/MS [6], twelve markers were preselected that represent the above dominant components of extracts. In the first step, the lacking structural information of two analytes was completed by isolation and subsequent spectroscopic studies (1D and 2D NMR). A quantitative profile of the extracts was then established using a RP-HPLC-PDA method, validated accordingly with the use of all selected markers as calibration standards. Next, the antioxidant activity of the markers was assessed in vitro in a biological model of human blood plasma exposed to oxidative stress, and in chemical tests (DPPH, FRAP and TBARS). Moreover, the synergistic effects between the representatives of the three main groups of *Cotoneaster* leaf polyphenols (flavanols, flavonols and caffeoylquinic acids) were evaluated in a selected model (FRAP). The combined data allowed for the defining of active markers, and for the proposing of a quality control approach that might be used in further studies on the extracts or for their therapeutic application.

## 2. Materials and Methods

### 2.1. Plant Material

The leaf samples of *Cotoneaster bullatus* Bois and *Cotoneaster zabelii* C.K. Schneid. were collected and authenticated in September 2015 in the Botanical Garden (51°45′ N 19°24′ E) in Lodz (Poland). Voucher specimens (KFG/15/CBL and KFG/15/CZB) were deposited in the Herbarium of the Department of Pharmacognosy, Medical University of Lodz (Poland).

### 2.2. General 

HPLC grade standards such as quercetin dehydrate (QU), chlorogenic acid hemihydrate (CHA), neochlorogenic acid (NCHA), cryptochlorogenic acid (CCHA), hyperoside semihydrate (HP), isoquercitrin (IQ), quercitrin (QR), rutin trihydrate (RT), ascorbic acid (AA) and Trolox^®^ (TX) were purchased from Sigma-Aldrich (Seelze, Germany/St. Louis, MO, USA). The standards of procyanidins B2 (PB2) and C1 (PC1), as well as (–)-epicatechin (ECA) were purchased from PhytoLab (Vestenbergsgreuth, Germany). Peroxynitrite (ONOO^−^) was synthesized according to [10]. HPLC grade solvents as acetonitrile, methanol and formic acid were from Avantor Performance Materials (Gliwice, Poland). All other reagents used in biological activity studies were of analytical grade and of the same origin as described elsewhere for the relevant tests [6,7]. 

Preparative column chromatography (CC) was performed using polyamide SC6 (Macherey -Nagel, Düren, Germany) and Sephadex LH-20 (Sigma-Aldrich, Seelze, Germany/St. Louis, MO, USA). Flash CC was carried out on VersaFlash HTFP system (Supelco, Sigma-Aldrich, Seelze, Germany/St. Louis, MO, USA) equipped with high-flow VersaFlash Piston Pump. The isolation process was carried out using a VersaPak C18 Cartridge (150 × 40 mm, Supelco, Sigma-Aldrich, Seelze, Germany/St. Louis, MO, USA). Analytical TLC was carried out on silica gel 60 G precoated plates (Merck, Darmstadt, Germany) using the horizontal DC chambers (Chromdes, Lublin, Poland) and the elution systems S-1 (EtOAc-HCOOH-H_2_O, 18:1:1, *v*/*v*/*v*) or S-2 (EtOH-25% NH_4_OH-H_2_O, 20:1:4, *v*/*v*/*v*).

### 2.3. Extracts Preparation 

The leaf samples of *C. bullatus* and *C. zabelii* were air-dried under normal conditions (20 °C, five days), powdered with an electric grinder, and sieved through a ø 0.315 mm sieve. The weighed samples (879 and 727 g, respectively) were first separately pre-extracted with chloroform (a Soxhlet apparatus) and then refluxed with methanol–water, 7:3, *v*/*v* (3 × 4 L, 8 h). The combined hydroalcoholic extracts were evaporated to dryness and lyophilized to obtain 31.7 g of *C. bullatus* leaf extract (MEB) and 38.6 g of *C. zabelii* leaf extract (MEZ). Portions of the extracts were left for phytochemical profiling and the studies of biological activity. The rest (25 g each) was resolved in hot water, cooled and the ballast precipitate was filtered off. The remaining filtrate (aqueous solution) was subsequently partitioned among solvents of rising polarity to give fractions of diethyl ether (DEF), ethyl acetate (EAF), *n*-butanol (BF) and the water residue (WR).

### 2.4. Isolation of Polyphenols from Cotoneaster Leaf Extracts 

The BF extract of *C. bullatus* leaves (10.2 g) was subjected to CC on Sephadex LH-20 with methanol as an eluent to give five fractions (F1–F5). Fraction F3 (0.20 g), after separation on a C18 flash column eluted with the gradient (20–60%; *v*/*v*) of methanol in 0.1% (*w*/*w*) aqueous formic acid at the flow rate 20 mL/min, gave compound QPH (299 mg). Fraction F4 (0.25 g) was crystallized from methanol to yield HP (35 mg). 

The EAF extract of *C. zabelii* leaves (18.1 g) was subjected to CC on polyamide and eluted successively with benzene-methanol mixtures with increasing proportion of methanol (up to 100%; *v*/*v*) to give fractions F1–F10. Fractions F3–F5 (0.35–0.50 g) were separately crystallized from methanol to yield compound HP (45, 35 and 22 mg, respectively). Fraction F4 (0.40 g) was then separated on a C18 flash column, eluted by ethyl acetate saturated by 0.1% (*w*/*w*) aqueous formic acid (flow rate 5 mL/min), to give after final purification over Sephadex LH-20 (eluted with methanol) compound QR (29 mg). Fractions F7 (1.15 g) and F9 (0.65 g) were separately re-chromatographed on Sephadex LH-20 with methanol to yield CHA (25 mg) and ECA (40 mg) from F7 as well as PB2 (80 mg) and CAD (20 mg) from F9. 

### 2.5. Structure Elucidation

#### 2.5.1. Acid Hydrolysis and Absolute Configuration of Monosaccharide Units in QPH

For total acid hydrolysis, QPH (3 mg) was refluxed for 1.5 h at 90 °C with 10% (*w*/*w*) aqueous HCl (3 mL). The reaction mixture, after cooling, was extracted four times with diethyl ether (3 mL). Sugars were identified in the aqueous layer by TLC (S-2 elution system) with authentic samples and detected with aniline phthalate at 105 °C. The absolute configuration of the monosaccharide moieties was determined according to Olszewska and Roj [11] by HPLC-PDA analysis of aqueous layer (the mobile phase: 40% acetonitrile in water (isocratic elution); V = 1 mL/min, column temperature 30 °C, detection wavelength λ = 230 nm) after conversion of the sugars to the 1-[(S)-*n*-acetyl-α-methylbenzylamino]-1-deoxy-aldiol-pentaacetate derivatives. The derivatives of authentic standards, obtained analogously, were used as references: derivative of d-xylose (t_R_ 15.60 min) and d-galactose (t_R_ 17.10 min). As the result, d-xylose and d-galactose were identified in the hydrolysate of QPH. 

#### 2.5.2. NMR Analysis

NMR spectra (^1^H, ^13^C NMR, ^1^H-^1^H COSY, HMBC and HMQC) were acquired in CD_3_OD or DMSO-d_6_ at 25 °C using a Bruker Avance III 600 MHz NMR spectrometer (Bruker BioSpin, Rheinstetten, Germany) and TMS as an internal standard. 

#### 2.5.3. Quercetin 3--*O*-β-d-(2″--*O*-β-d-xylopyranosyl)galactopyranoside (QPH)

Yellow needles. HPLC: t_R_ = 9.57 min. ^1^H NMR (600 MHz, DMSO-d_6_): δ 12.70 (s, 1H, OH-5), 7.76 (dd, J = 2.3, 8.7 Hz, 1H, H-6′), 7.53 (d, J = 2.3 Hz, 1H, H-2′), 6.84 (d, J = 8.7 Hz, 1H, H-5′), 6.40 (d, J = 1.9 Hz, 1H, H-8), 6.20 (d, J = 1.9 Hz, 1H, H-6), 5.70 (d, J = 7.5 Hz, 1H, H-1″), 4.57 (d, J = 7.2 Hz, 1H, H-1‴), 3.78 (dd, J = 7.9, 9.4 Hz, 1H, H-2″), 3.72 (dd, J = 5.3, 11.9 Hz, 1H, H-5a‴), 3.70 (brd, J = 3.0 Hz, 1H, H-4″), 3.63 (dd, J = 3.4, 9.4 Hz, 1H, H-3″), 3.44 (dd, J = 6.4, 10.9 Hz, 1H, H-3‴), 3.37 (t, J = 6.4 Hz, 1H, H-6a″), 3.27 (m, 2H, H-4‴, H-6b″), 3.16 (t, J = 8.3 Hz, 1H, H-2‴), 3.09 (t, J = 7.5 Hz, 1H, H-5″), 3.09 (dd, J = 10.9, 11.3 Hz, 1H, H-5b‴). ^13^C NMR (600 MHz, DMSO-d_6_): 177.9 (C-4), 164.5 (C-7), 161.8 (C-5), 156.7 (C-2), 155.8 (C-9), 149.0 (C-4′), 145.4 (C-3′), 133.6 (C-3), 122.7 (C-6′), 121.7 (C-1′), 116.3 (C-5′), 115.7 (C-2′), 105.1 (C-1‴), 104.4 (C-10), 99.1 (C-6), 98.9 (C-1″), 93.9 (C-8), 80.3 (C-2″), 76.7 (C-3‴), 76.4 (C-5″), 74.41 (C-2‴), 74.1 (C-3″), 69.9 (C-4‴), 68.3 (C-4″), 66.1 (C-5‴), 60.5 (C-6″).

#### 2.5.4. Caffeoylmalic Acid (CAD)

White needles. HPLC: t_R_ = 9.12 min. ^1^H NMR (600 MHz, CD_3_OD): δ 7.61 (d, J = 15.8 Hz, 1H, H-7), 7.08 (d, J = 1.9 Hz, 1H, H-2), 6.98 (dd, J = 8.3, 1.9 Hz, 1H, H-5), 6.80 (d, J = 8.3 Hz, 1H, H-6), 6.34 (d, J = 15.8 Hz, 1H, H-8), 5.46 (dd, J = 9.0, 3.8 Hz, 1H, H-2′), 2.98 (dd, J = 16.4, 3.8 Hz, 1H, H-3a′), 2.86 (dd, J = 16.4, 9.0 Hz, 1H, H-3b′). ^13^C NMR (600 MHz, CD_3_OD): 172.0 (C-1′, C-4′), 167.0 (C-9), 148.2 (C-4), 146.0 (C-3), 145.4 (C-7), 126.5 (C-1), 121.7 (C-6), 115.2 (C-5), 113.8 (C-2), 113.5 (C-8), 69.8 (C-2′), 36.5 (C-3′).

### 2.6. Quantitative HPLC-PDA Assay and Method Validation

The HPLC-PDA assays were carried out on a Waters 600E Multisolvent Delivery System (Waters, Milford, MA, USA) with a PDA detector (Waters 2998); a manual 7725 sample injection valve with a 5 µL injection loop (Rheodyne, Pittsburgh, PA, USA). A C18 Ascentis^®^ Express column (2.7 µm, 75 × 4.6 mm i.d.; Supelco, Bellefonte, PA, USA) with a C18 Ascentis^®^ C18 Supelguard column (3 µm, 20 × 4 mm i.d.; Supelco, Bellefonte, PA, USA) were maintained at 25 °C using a Jetstream Plus 5480 thermostat (Thermotechnic Products, Langenzersdorf, Austria). The mobile phase consisted of solvent A (0.5% orthophosphoric acid in water, *w*/*v*) and solvent B (acetonitrile) with the elution profile: 0–14 min, 6%–30% B (*v*/*v*), 14–15 min, 30%–50% B; 15–17 min, 50% B; 17–18 min, 50%–6% B; 18–21 min, 6% B (equilibration). The flow rate was set at 1.4 mL/min. Before analyses, samples of the extracts were dissolved to 2.0–2.5 mg/mL in methanol–water (7:3 *v*/*v*), and filtered through a syringe PTFE filter (25 mm, 0.22 μm; Alchem, Toruń, Poland). The UV-vis data were collected in the range 220–450 nm and the polyphenols were classified according to their spectra. The λ for detection and quantification was set to 280 nm for flavanols, 325 nm for caffeic acid derivatives, and 350 nm for flavonoids. Twelve external standards (ECA, PB2, PC1, CAD, CHA, NCHA, CCHA, QPH, RT, HP, IQ, QR) were used for calibration. The tentatively identified peaks were quantified as equivalents of ECA (flavanols), CHA (caffeic acid derivatives) and HP (flavonoid glycosides).

The proposed HPLC-PDA method was validated by the determination of the selectivity, linearity, precision and accuracy of the analytes, according to the ICH Guidance for Industry [12] and some previous literature reports [13]. The standard stock solution of twelve reference polyphenols was prepared in methanol–water (7:3, *v*/*v*) and serially diluted with the same solvent to at least six concentration levels. The replicate solutions were injected triply into the HPLC system and the calibration graphs were constructed by plotting the mean peak area versus concentration. The limits of detection (LOD) and limits of quantification (LOQ) were determined based on a series of dilutions of the stock solution with methanol–water (7:3, *v/v*) to obtain the 3-σ signal-to-noise (S/N) ratio for an LOD and S/N greater than 10 for LOQ values. The precision was tested on the basis of the *RSD* values for peak areas and retention times obtained for two concentration levels of the standard solution (at 25% and 75% of the highest linear range limits). The intra-day variability was established by triplicate analysis of each sample on the same day within 24 h. The inter-day reproducibility was determined on three different days with triplicate injection of each sample during each of the three days. To evaluate the accuracy, a recovery test was performed in the *C. bullatus* leaf extract spiked with two different concentration levels of each standard (at 10% and 25% of the highest linear range limits). The accuracy was calculated as the mean recovery of the standards from the spiked extract solutions versus the non-spiked extract samples.

### 2.7. Antioxidant Activity in Chemical Models

The DPPH free-radical scavenging activity was determined according to the previously optimized method [14] and expressed as SC_50_ values (μg/mL, μmol/L) calculated from concentration–inhibition curves. The FRAP was assayed as described previously [14] and expressed in mmol of ferrous ions (Fe^2+^) produced by 1 g of the analytes or extracts, as calculated from the calibration curve of ferrous sulfate. The ability of the phenolics to inhibit AAPH-induced peroxidation of linoleic acid (LA) was assayed as described previously [7] with the peroxidation monitored by quantification of thiobarbituric acid-reactive substances (TBARS), and results were expressed as IC_50_ values (μg/mL, μmol/L) calculated from concentration–inhibition curves. Additionally, the activity parameters of all of the assays were expressed as mmol Trolox equivalents (TE) per g weight of the analytes or extracts (mmol TE/g dw). 

### 2.8. Antioxidant Activity in Human Plasma Model

Human blood buffy coats (from eight healthy volunteers) were purchased from the Regional Centre of Blood Donation and Blood Treatment in Lodz (Poland). The blood plasma samples were obtained by centrifugation according to previously described procedures [15]. All experiments were accepted by the committee on the Ethics of Research at the Medical University of Lodz RNN/347/17/KE. Plasma samples were pre-incubated for 15 min at 37 °C with the examined phenolics/extracts, added to a final concentration range of 1–50 µg/mL, and then exposed to 150 µM (FRAP assay) or 100 µM of ONOO^−^ (the remaining experiments on blood plasma). Control samples were prepared with plasma untreated by the analyte and/or ONOO^−^. The 3-nitrotyrosine-containing proteins were detected by the competitive ELISA test (enzyme-linked immunosorbent assay), as described previously [16]. The concentrations of nitrated proteins were estimated from the standard curve of 3-nitrotyrosine-containing fibrinogen (3-NT-Fg) and expressed as the 3NT-Fg equivalents (in nmol/mg of plasma proteins). The concentration of free -SH groups in plasma samples was measured spectrophotometrically according to the Ellman′s method, as described earlier [15], and -SH concentrations were expressed in µmol/mL of plasma. The TBARS levels were determined as described previously [16] and expressed in μmol TBARS/mL of plasma. NEAC of plasma was evaluated by measuring its ferric-reducing ability (FRAP), as described by Marchelak et al. [16], and the results were expressed in mM of Fe^2+^ equivalents. 

### 2.9. Contribution of Individual Polyphenols to Antioxidant Activity of Cotoneaster Leaf Extracts 

The percentage contribution of polyphenols (*CT*) to the activity of the investigated extracts (MEB, MEZ) was calculated based on the FRAP results (in chemical and biological models), according to Equation (1)

(1) $$CT\left(\%\right)=\frac{C_{P}\times {\mathrm{AE}}_{P}}{{\mathrm{AE}}_{E}}\times 100\%$$

where C*_P_* is the content (g/g dw) of an analyte in the MEB or MEZ as presented in Table 2; AE*_P_* is the activity of the analyte expressed in the AA equivalents (g/g dw); AE*_E_* is the activity of the given extract (MEB or MEZ) expressed in AA equivalents (g/g dw). 

The *CT*s for the groups of phenolics were calculated as the sum of the contributions of individual analytes belonging to that group. In the case of the analytes whose activity was not tested directly, AE*_P_* values of the model compound for a particular group were used (HP for flavonoids, CHA for caffeic acid derivatives and PC1 for flavanols). The *CT* for TPA was obtained using their content, obtained in the previous study [6], and the AE_P_ value for PC1.

### 2.10. Interaction Effects of Combined Polyphenols

The interactions between the representatives of the main extracts constituents (ECA, QPH, CHA) were studied in the chemical FRAP assay (according to Section 2.7) using an isobolographic analysis and by calculating interaction factors (*IF*) for two-component mixtures of the selected compounds [17,18].

First, the reduction potential of the three pure compounds was tested at different concentration levels and five-point dose–response curves were constructed (linear regression model). Using the curves, effective concentrations (*EC*) of the substances were calculated, that were defined as the concentrations required for a reduction in 34 µM Fe^3+^ (corresponding to the absorbance of 0.5) under the conditions of the experiment. The obtained *EC* values served as the basis for plotting additivity isoboles (lines that indicate the points representing the dose-combinations of the two analytes that give the expected effect, considering no interaction). 

In the next step, two-component mixtures were prepared at five different concentration ratios (1:4, 1:2, 1:1, 2:1, and 4:1), and for each mixture the *EC* value was calculated using a five-point dose–response curve (individually constructed for each concentration ratio). The values were then plotted on the isobologram. The points that fall on the additivity isobole indicate no interaction, while those below or above suggest synergy or antagonism, respectively.

The type of interaction between the studied analytes was also assessed through the determination of an interaction factor (*IF*), calculated by Equation (2)

(2) $$IF=\frac{C_{E1}}{C_{T1}}+\frac{C_{E2}}{C_{T2}}$$

where C_*E*1_ and C_*E*2_ are the concentrations of the analytes in the two-component mixture, producing the expected FRAP effect (calculated from the experimentally determined *EC* value for the mixture); while C_*T*1_ and C_*T*2_ are the theoretical concentrations of the analytes producing the expected effect, calculated from the individual dose–response curves of the analytes. An *IF* equal to one indicated additivity, while an *IF* less then or more than one indicated synergy and antagonism, respectively. For each dose combination, the experiments were run in triplicate. 

### 2.11. Statistical Analysis 

The results are presented as mean ± standard deviation (SD), or ± standard error (SE), for the indicated number of experiments. The significance of differences between means was determined using one-way ANOVA followed by the post-hoc Tukey′s test (quantitative analysis and antioxidant activity in chemical models), or one-way ANOVA for repeated measures followed by Dunnett′s tests (antioxidant activity in biological models). All calculations were performed using Statistica 12 Pl software for Windows (StatSoft Inc., Krakow, Poland) with *p* values less than 0.05 regarded as significant. 

## 3. Results and Discussion

### 3.1. Selection of Potential Active Markers 

**Figure 1:**
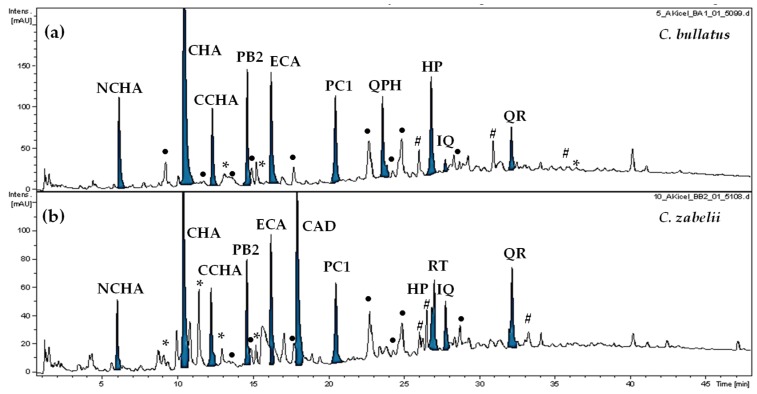
Representative UHPLC-UV chromatograms of the methanol-water (7;3, *v*/*v*) extracts from the leaves of *C. bullatus* (**a**) and *C. zabelii* (**b**) at 280 nm. Potential active markers are marked in blue. NCHA, neochlorogenic acid (3--*O*-caffeoylquinic acid); CHA, chlorogenic acid (5--*O*-caffeoylquinic acid); CCHA, cryptochlorogenic acid (4--*O*-caffeoylquinic acid); CAD, caffeic acid derivative; ECA, (–)-epicatechin; PB2, procyanidin B2; PC1 procyanidin C1; QPH, quercetin pentoside-hexoside; RT, rutin; HP, hyperoside; IQ, isoquercitrin; QR, quercitrin. Other identified constituents are labeled with the appropriate symbol depending on the phytochemical group they belong to: •, B-type procyanidins; *, caffeic and *p*-coumaric acids derivatives; **#**, quercetin glycosides. For details of compounds′ identification, see Supplementary Materials, Figure S1 and Table S1.

The extraction process is important to obtain the representative polyphenolic profile of a studied plant material. For our investigation, heating reflux was selected for this task, as it is superior to new methods such as ultrasonic and microwave-assisted extraction, mainly due to the demonstrated decomposition of polyphenolic molecules by reactive oxygen species and/or ultra-high temperatures during sonication or irradiation [19]. Additionally, as previously shown [5,6], methanol–water (7:3, *v*/*v*) was the optimal solvent, giving a high content of *Cotoneaster* polyphenols and high extraction yields. In particular, our previous study on *C. bullatus* and *C. zabelii* [6] revealed that the methanol–water extracts (7:3, *v*/*v*) are rich in low-molecular weight polyphenols, including caffeic acid derivatives, mono- and oligomeric flavan-3-ols, and flavonoids (more than thirty molecules fully or tentatively identified by UHPLC-PDA-ESI-MS^3^; for details see Supplementary Material, Table S1 and Figure S1). Polyphenols representing all of these classes of compounds are known for their relevant antioxidant properties, thus, twelve dominating representatives of these groups were preselected as potential active markers (Figure 1). Ten of them were identified previously by comparison of their chromatographic and MS/MS spectral data with those of authentic standards [6]. Among them were: 5--*O*-caffeoylquinic acid (chlorogenic acid, CHA), 3--*O*-caffeoylquinic acid (neochlorogenic acid, NCHA), 4--*O*-caffeoylquinic acid (cryptochlorogenic acid, CCHA), (–)-epicatechin (ECA), procyanidin B2 (PB2), procyanidin C1 (PC1), rutin (RT), hyperoside (HP), isoquercitrin (IQ) and quercitrin (QR). Identification of the remaining two, assigned as quercetin pentoside-hexoside (QPH) and caffeic acid derivative (CAD), required isolation and detailed structural elucidation.

Moreover, according to the previous studies [6], the extracts contain also an abundant fraction of high-molecular-weight proanthocyanidins, which, due to low bioavailability, might be of lesser importance to the potential systemic activity of the extracts. On the other hand, the antioxidant potential of those phenolics may have meaning when it comes to the activity within the gastrointestinal tract or topical applications related to its antimicrobial or anti-hemorrhagic properties [20]. Thus, their contribution to the activity of the target extracts should not be neglected.

### 3.2. Isolation and Identification of Cotoneaster Polyphenols

CAD and QPH were obtained from the methanol–water (7:3, *v*/*v*) leaf extracts of *C. bullatus* and *C. zabelii*, respectively, after fractionation and chromatographic separation using open (polyamide SC6, Sephadex LH-20) and flash (C18) column chromatography. The structures of the isolates were determined based on 1D and 2D NMR experiments (^1^H and ^13^C NMR, COSY, HMBC and HMQC), and the absolute configuration of sugars was confirmed by RP-HPLC analysis of the acid hydrolysates.

**Figure 2:**
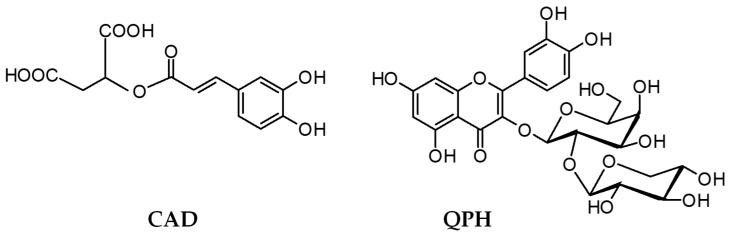
Structures of the isolated phenolics—caffeoylmalic acid (CAD) and quercetin 3-*O*-(2″-*O*-β-D-xylopyranosyl)-β-d-galactopyranoside (QPH).

Compound QPH was based on MS data classified as quercetin pentoside–hexoside [6]. The HPLC analysis of the acid hydrolysis products allowed for the identification of d-galactose and d-xylose as the sugar components. The ^1^H NMR spectrum of QPH revealed five signals attributable to the aglycone part that corresponded to the five aromatic protons of quercetin. The two anomeric protons of the sugar moieties gave signals at δ 5.70 and δ 4.57 ppm with coupling constants of 7.5 and 7.2 Hz, respectively, indicating β-glycosidic bonds and pyranose form of the galactose and xylose units [21]. The HMBC correlations of those resonances allowed, in turn, for the establishment of the attachment positions of the sugar moieties. Consequently, in accordance with the literature data [21,22], QPH was identified as quercetin 3-*O*-(2″-β-d-xylopyranosyl)-β-d-galactopyranoside (Figure 2).

Compound CAD, based on its UV- and MS-spectra, was previously speculated to be a caffeic acid derivative [6]. Indeed, the ^1^H and ^13^C NMR spectra exhibited signals typical of a caffeoyl residue. The three additional proton signals at δ 2.98 (1H, dd, J = 16.4, 3.8 Hz), 2.86 (1H, dd, J = 16.4, 9.0 Hz) and 5.46 ppm (1H, dd, J = 9.0, 3.8 Hz), and the carbon resonances at δ 172.0 (2C), 69.8 and 36.5 ppm in combination with the respective 2D NMR data, were resolved as coming from malic acid. The HMBC correlation between H-2′ (δ 5.46 ppm) and C-9 (δ 167.0 ppm) determined the position of the ester bond and allowed for the eventual identification of CAD as caffeoylmalic acid (Figure 2) [23,24]. 

QPH has previously been isolated from several taxa, e.g., *Trifolium repens* flowers, *Saxifraga stolonifera* aerial parts, *Ocimum lamiifolium* leaves, and *Armoracia rusticana* leaves [21,22,25,26], while CAD was obtained for the first time from *Raphanus sativus* cotyledons [23] and since then was found, among others, in *Ballota larendana* aerial parts, *Lactuca sativa* leaves and *Brassica rapa* leaves [24,27,28]. Considering their relatively rare natural occurrence, both compounds may be of particular importance for the *Cotoneaster* genus from a chemotaxonomic point of view. They may also differentiate individual species within the genus. For instance, during our previous screening tests [5] QPH and CAD were found in the leaves of only six and four of twelve *Cotoneaster* species studied, respectively.

In the present study, in addition to CAD and QPH, five further *Cotoneaster* polyphenols were separated during the isolation process. Among them were CHA, ECA, PB2, HP and QR. Their structures were unequivocally confirmed based on a direct comparison of the chromatographic and a wide range of spectroscopic data (ESI-MS, 1D and 2D NMR) with those of authentic standards or the data given in the literature [29,30,31,32,33,34]. To our knowledge, this is the first report on the isolation of CHA, CAD, ECA, PB2, QR and HP from *C. zabelii*, - and QPH and HP from *C. bullatus*. 

### 3.3. Quantification of Phenolics 

**Figure 3:**
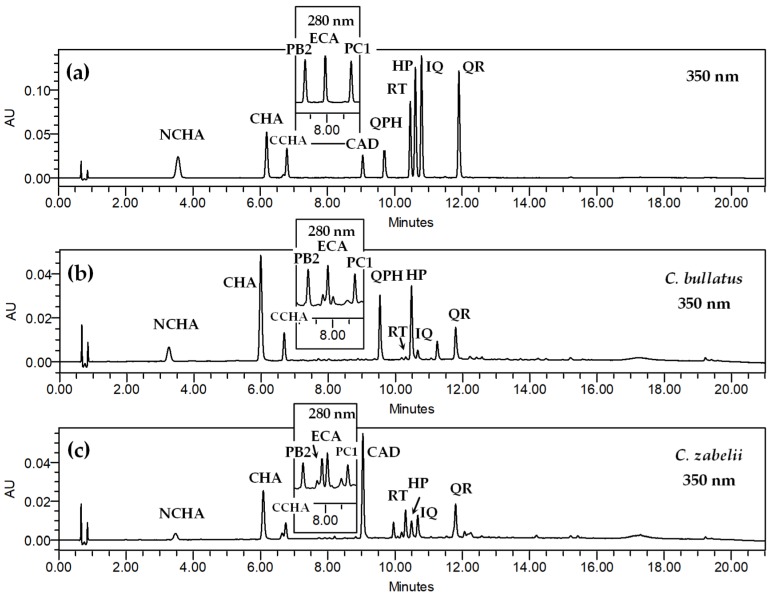
Representative HPLC-UV chromatograms of the phenolic standards (**a**) and the methanol–water (7:3, *v*/*v*) extracts from the leaves of *C. bullatus* (**b**) and *C. zabelii* (**c**) at 350 and 280 nm. For compound codes, see Figure 1.

The content of the low-molecular weight phenolics in the investigated extracts was determined by RP-HPLC-PDA method. The applied procedure was optimized to satisfactorily separate the plant matrix (Figure 3), and the use of a fused-core column technology shortened the time of the analysis to 21 min, in comparison to the 63 min achieved in the qualitative UHPLC method [7].

Routine application of the method for the purpose of quality assurance required confirmation and the provision of objective evidence, so that the analytical results might be obtained with an acceptable uncertainty level and the proposed analytical procedure fits with the specified intended use [35]. The developed method was, thus, fully validated according to ICH Guidance for Industry [12] and the obtained parameters of linearity, sensitivity, precision and accuracy (Table 1) indicated its suitability for the defined task.

However, the optimization tests showed that, due to the gradient elution, the separation process was relatively susceptible to variations in mobile phase composition, flow rate and column temperature. Therefore, a series of system suitability parameters, such as peak resolution (*R_s_*), symmetry factors (*T*) and *RSD* values for retention times, should be regularly controlled with the use of standard solution to ensure that the validity of the method is maintained whenever used. According to the experimental data (Supplementary Materials, Table S2), the *R_s_* values of the critical, closely eluting peak pairs of RT/HP and HP/IQ should be primarily monitored for that purpose.

The developed method was successfully applied for the quantification of the dominant polyphenols in *Cotoneaster* extracts in this and previous studies [6]. Additionally, the minor constituents of the extracts were classified according to their UV-spectra to one of the three main classes of polyphenols and were quantified as equivalents of ECA (flavanols), RT and HP (flavonoids) or CHA (caffeic acid derivatives), accordingly. This allowed for the evaluation of total low-molecular proanthocyanidins (TLPA), total flavonoids (TFL) and total caffeic acid derivatives (TCA) content, as well as total content of low-molecular weight phenolic (TPH) in the extracts.

According to the results (Table 2), the TPH levels in the investigated extracts reached 96.1 and 102.0 mg/g of the dry weight for *C. bullatus* and *C. zabelii*, respectively. About 85% of those values can contribute to the twelve selected markers, which illustrates their dominant role in the extracts. In the extract of *C. bullatus*, the low-molecular flavanols (TLPA) were the major group of phenolics constituting over 50% of the TPH level. In addition to the high contents of PB2, PC1 and ECA, the extract also contained relevant amounts of CHA. On the other hand, in the extract from *C. zabelii*, caffeic acid derivatives prevailed, and their total levels (TCA) accounted for over 45% of the TPH, with CAD being the clearly dominating constituent. Still, the TLPA values in that extract were also relevant (almost 40% of TPH). In both extracts, the levels of flavonoids (TFL) contributed the least to the TPH contents, with the representatives of mono- and diglycosides, HP and QPH for *C. bullatus* and QR and RT for *C. zabelii*, being the prevailing constituents.

### 3.4. Antioxidant Activity of the Selected Markers in Chemical Models

To assess the basic antioxidant potential of the preselected markers, their activity was tested in chemical in vitro models based on single electron transfer (DPPH, FRAP) and hydrogen atom transfer (TBARS), to study the interactions of the compounds with free-radicals (DPPH) and transition metal ions (FRAP) and to evaluate their protective effects against lipid peroxidation (TBARS). All investigated polyphenols displayed dose-dependent activity in a range similar to that of a series of known antioxidant standards, quercetin (QU), Trolox^®^(TX), and ascorbic acid (AA) (Table 3). In all of the employed tests, the flavan-3-ol derivatives (ECA, PB2, PC1) were the most active antioxidants. When expressed in weight units, their potency was at a similar level, as evidenced by the values of DPPH SC_50_ (2.4–2.6 µg/mL) and TBARS IC_50_ (2.3–2.9 µg/mL). On the other hand, comparing their molar activity (µmol/L, DPPH, TBARS), the oligomers (PB2 and PC1) were found to be superior (DPPH, SC_50_ = 4.3 and 2.9 µmol/L, respectively) than the monomer (ECA; DPPH, SC_50_ = 8.1 µmol/L). This fact is in agreement with previous results [36,37], showing that the degree of polymerization of flavanols increases their effectiveness against radical species. As for caffeic acid derivatives (CHA, CAD), their relatively high antioxidant activity is associated mainly with the presence a catechol-type ring moiety and conjugated double bonds. Such molecular structure enables, among others, the effective delocalization of unpaired electrons [38].

In the case of the investigated flavonoids, their activity in all tests in weight units (Table 3) decreased in the order aglycone QU > monoglycosides of QU (HP, IQ and QR) > diglycosides of QU (RT and QPH). This visible effect of the structure–activity relationships is in accordance with the proven fact that the glycosylation of flavonoids reduces their activity compared to the corresponding aglycones [39]. However, when the antioxidant parameters were expressed in µmol/L (DPPH, TBARS) or mol/mol (FRAP), the differences in activity were much less pronounced, and mono- and diglycosides were proven to be comparable to, or even more active than, the QU. This indicates that the catechol moiety and phenol groups in A-ring of a flavonoid skeleton might have a greater impact on the antioxidant activity of flavonoids, than, as generally suggested, the free C-3 hydroxyl group.

### 3.5. Antioxidant Activity of the Selected Markers in Human Plasma Model

**Figure 4:**
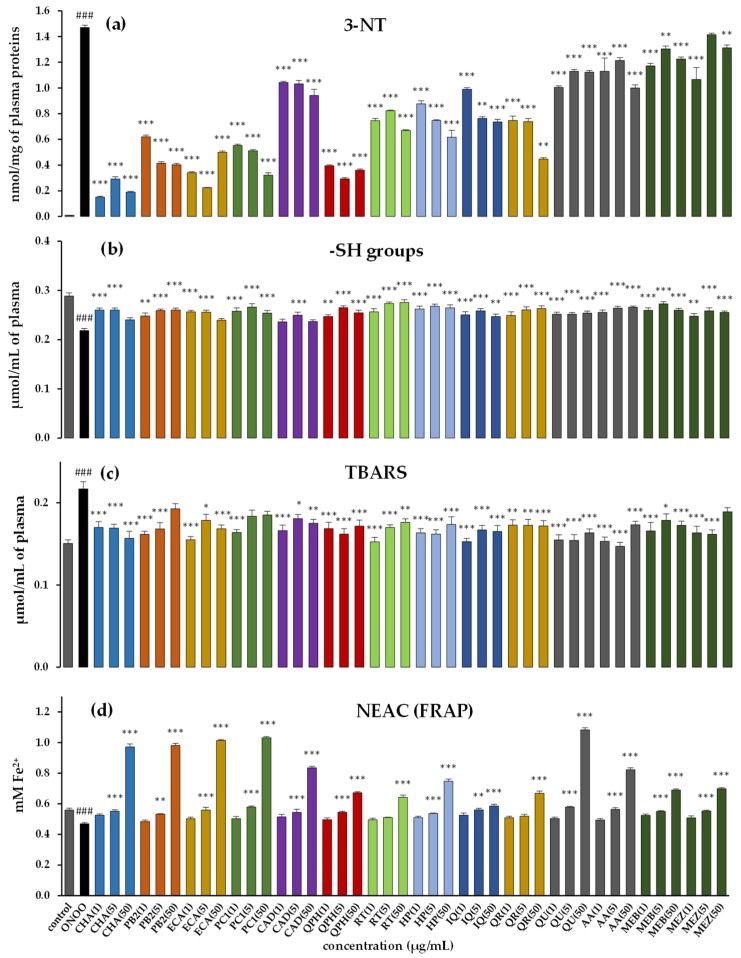
Effects of *Cotoneaster* phenolics on human plasma exposed to oxidative stress: (**a**) effects on the nitration of tyrosine residues in plasma proteins and formation of 3-NT; (**b**) effects on the oxidation of free -SH groups; (**c**) effects on the peroxidation of plasma lipids and formation of TBARS; (**d**) effects on the non-enzymatic capacity of plasma (NEAC), assessed by the FRAP assay. Results expressed as means ± SE (*n* = 8) for repeated measures: ### *p* < 0.001, for ONOO^−^-treated plasma (without the analytes) versus control plasma, and *** *p* < 0.001, ** *p* < 0.01, * *p* < 0.05 for plasma treated with ONOO^−^ in the presence of the investigated markers, extracts or standards (1–50 µg/mL) versus ONOO^−^-treated plasma in the absence of the analytes. For compounds and extract codes, see Figure 1 and Table 2. Positive controls: QU, quercetin; TX, Trolox^®^; AA, ascorbic acid.

In the next step, the antioxidant effects of the preselected compounds were evaluated using the more complex and in vivo-relevant model of human plasma exposed to oxidative stress induced by peroxynitrite (ONOO¯). This powerful oxidative and nitrative species is generated in vivo by the reaction of nitric oxide (NO) and superoxide anion (O_2_•^−^), and is involved in the pathophysiology of various inflammatory, neurodegenerative and cardiovascular disorders [40]. Evidence for the harmful action of ONOO¯ can be seen in protein and lipid modifications reflected, i.a., in the levels of 3-nitrotyrosine (3-NT) and thiol groups (markers of protein nitration and oxidation), as well as the level of TBARS (markers of lipid peroxidation). As demonstrated (Figure 4a–d), the exposure of plasma samples to 100 or 150 µM ONOO¯ results in a range of oxidative and nitrative damage. In comparison to the control samples (untreated plasma), the ONOO¯-treated plasma exhibited a considerably enhanced level (*p* < 0.001) of 3-NT in plasma proteins, with, at the same time, a reduced level of thiol groups (–SH). Moreover, an increase (*p* < 0.001) in TBARS and decrease (*p* < 0.001) in the non-enzymatic antioxidant capacity (NEAC) of plasma measured by the FRAP assay were observed.

The studied plasma samples were simultaneously incubated with the selected polyphenols as well as the *Cotoneaster* extracts. The applied wide concentration range (1–50 µg/mL) included the levels of antioxidants potentially achievable in vivo (1–5 µg/mL) after oral administration of polyphenol-rich products [41,42,43]. In the samples incubated with the model polyphenols or extracts, the range of the oxidative/nitrative alterations of the plasma proteins and lipids was visibly diminished (*p* < 0.05). A statistically significant (*p* < 0.05) decrease in tyrosine nitration (by about 29–90%) was found for all investigated polyphenols, but the highest activity of ECA, CHA and QPH was clearly noticeable (Figure 4a). The protective effects on the thiol groups were less diversified, and all polyphenols, regardless of the employed concentration, displayed statistically significant efficacy in the narrow range of 12–20%, with the exception of the higher concentrations (50 µg/mL) of ECA, CAD and CHA, which were less potent this time (Figure 4b). The investigated polyphenols were also able to protect plasma lipids against ONOO¯-induced peroxidation, as demonstrated by the diminished (by about 15–25%) levels of TBARS (Figure 4c). Moreover, the polyphenols, especially at the higher concentration (5–50 µg/mL), increased the plasma reducing ability by up to 57% versus the ONOO¯-treated plasma (Figure 4d). 

These findings may indicate that the studied polyphenols are responsible for the biological effects of *Cotoneaster* leaves and might be regarded as active markers for the purpose of quality control. However, the observed antioxidant effects of *Cotoneaster* polyphenols in most of the biological tests (the exception was the protective effect against protein nitration) were comparable to that of the source hydromethanolic extracts. Considering the fact that the selected markers constituted only about 8–9% of the dry extracts, the antioxidant capacity of the extracts might be partly the result of the effects of other metabolites, including other polyphenols, or may be a consequence of the synergistic action between individual phenolics.

### 3.6. Contribution of Individual Polyphenols to Antioxidant Activity of the Extracts

To provide an insight into the contribution of individual polyphenols and groups of polyphenols to the activity of the *Cotoneaster* extracts, their theoretical shares in the extracts′ activity were estimated (Table 4). The contribution factors (*CT*) were calculated (Equation (1)) based on the content of particular phenolics in the extracts and FRAP results (in both chemical and biological models) expressed by the equivalents of ascorbic acid (AE). 

The total contributions of the twelve markers and the TPH fraction (including minor phenolic constituents) to the extracts activity was, depending on the extract and the analytical model, about 20%–27%, so higher that might be implied from their content (Table 4). Still, however, a large part of the extracts′ activity remained unexplained. As was found previously, the investigated extracts also contain a relatively high content of high-molecular-weight proanthocyanidins (TPA), which, however, could not be assessed by RP-HPLC methods. The contribution of that group of compounds was estimated based on their content, as evaluated previously [6] using the spectrophotometric method (about 24% dw, Table 2) and the activity of PC1. The obtained values of TPA′s contribution were around 50–60% (Table 4). It has to be remembered that this value might partly overlap with the share of TLPA, as the spectrophotometric *n*-butanol/HCl assay is not specific. Still, the high-molecular-weight proanthocyanidins could have a prominent role in the activity of the *Cotoneaster* extracts observed in the present study, and thus also in their biological efficacy.

### 3.7. Interactions of Model Polyphenols in the FRAP Assay

Due to the complex composition of plant products there is a high probability of interactions between their various constituents, that may be reflected in the overall activity both positively (synergy) and negatively (antagonism). In the case of the investigated *Cotoneaster* extracts, their activity was greater than predicted by the capacities of individual analytes, which suggested the possibility of some synergistic effects.

For a closer investigation of those effects, representatives of the three main classes of polyphenols, present in the leaves of *C. bullatus* and *C. zabelii* (flavanols, flavonoids and caffeic acid derivatives), i.e., ECA, QPH and CHA, were selected. The antioxidant capacities of the two-component combinations were investigated using the FRAP assay, in which the above polyphenols were characterized by their high antioxidant potential, both in the chemical test (Table 3) and in the model of human plasma (Figure 4).

**Figure 5:**
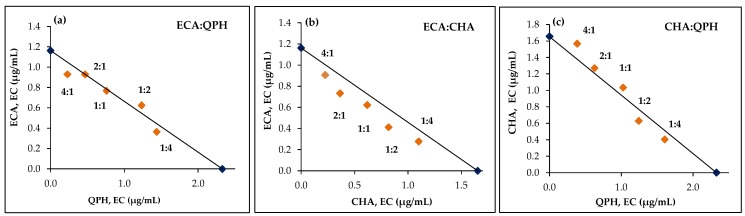
Effective concentration (EC) isobologram for the interactions between (**a**) ECA and QPH; (**b**) ECA and CHA; (**c**) CHA and QPH in the chemical FRAP model. The straight line is an estimated isobole for an additive interaction between two substances based on the *EC* of pure phenolics (dark blue datapoints). The orange datapoints represent the experimental *EC* values for combinations of the investigated analytes. The *EC* parameter indicates the concentration of an analyte required for a reduction in 34 µM of Fe^3+^ in the conditions of the experiment.

To investigate the interactions between the investigated compounds, an isobolographic analysis developed by Loewe was used [18]. First the concentrations of pure substances (effective concentrations, *EC*) required to exert a specified FRAP effect were determined (for details see Section 2.10). Based on those data, a theoretical isobole (a curve that connects points representing drug combinations that give same effect) was constructed, illustrating an additive effect of two substances (Figure 5). Then, the experimental *EC* for different combinations of the investigated substances were established, and the results were marked on the graph. The datapoints that fall below the additivity isobole suggest a synergistic effect, while those above suggest an antagonistic one (Figure 5). Additionally, interaction factors (*IF*) were calculated (Equation (2)) for quantitative evaluation of the observed effect. An *IF* equal 1 indicated additivity, while values significantly lower or greater than 1 suggested synergy and antagonism, respectively (Table 5). As interaction effects depend both on the properties and the exact dose of the individual analyte [44], each combination of the compounds was tested in three concentration ratios. The concentrations and proportions used in the tests were selected on the basis of the reducing power of individual compounds (Table 3) and on the relative proportions of these compounds in the source extracts (Table 2).

As indicated by the results (Figure 5, Table 5), the tested compounds exhibited different effects, depending on the combination. The best synergy might be observed between ECA and CHA, for which synergistic effects were observed regardless of the concentration ratio. For example, for a ECA:CHA (4:1) mixture, the *EC* was achieved at 0.95 µg/mL, while 1.16 and 1.65 µg/mL of each analyte separately was required for the same effect. In addition, ECA, one of the superior *Cotoneaster* antioxidants (Table 3), is also able to increase the antioxidant activity of flavonoids. The combination of ECA:QPH was especially effective at ratios of 4:1 and 1:4, for which a synergistic effect was obtained at total analytes concentrations of 1.18 and 1.80 µg/mL, respectively, compared to 2.33 µg/mL of pure QPH. In the case of the combination CHA:QPH, synergy was observed only if the flavonoid prevailed in the mixture.

Our findings are in accordance with the accumulated literature data on the synergistic effects between polyphenols, demonstrated in several analytical models. For example, an analysis of the synergy between ECA, CHA and IQ in the O_2_^•−^ scavenging test showed that the combination of CHA and IQ in the proportion of 1:2 µg/mL is optimal for achieving a 50% scavenging effect, while up to 4.4 µg/mL of pure CHA or 7.8 µg/mL of IQ was needed to obtain the same effect [45]. The interactions between CHA and RT as well as CHA and QU (the proportions in both cases were 0.6:0.15 µmol/mL) showed synergistic effects by increasing the FRAP value by up to 17.2% [46]. In addition, some other antioxidants, such as rosmarinic and gallic acids, have been also described to synergistically improve the antioxidant activity of CHA, QU and RT [46]. In the case of flavanols, not only do they exhibit synergy with exogenous polyphenols, known for their antioxidant properties, but they might also act synergistically with endogenous antioxidants [20]. This effect was observed in the DPPH test between (+)-catechin and glutathione, and it was proved that the presence of the catechol group in the B ring of the flavanol skeleton plays a crucial role in this type of interaction [47]. The synergism between individual polyphenols or groups of these compounds may not only improve the properties of a singular raw material or extract but also positively affect the antioxidant activity of their various combinations. For example, the antioxidant activity of the leaves of *Potentilla fruticosa*, containing mainly hydrolysable tannins, was synergistically increased by the flavonoid fraction of *Ginkgo biloba* leaves [48].

## 4. Conclusions

This study is the first evaluation of active markers of *Cotoneaster* leaves and their contribution to the antioxidant activity of the leaf extracts of *C. bullatus* and *C. zabelii*. Twelve analytes selected during the study represent the classes of polyphenols typical of the analyzed species, i.e., flavan-3-ols (ECA, PB2, and PC1), caffeic acid derivatives (CHA, NCHA, CCHA, and CAD), and flavonols (QPH, HP, QR, IQ, and RT), and their levels constitute about 85% of the total contents of low-molecular weight phenols in the extracts. Moreover, two of the markers (QPH and CAD) have high chemotaxonomic value and differentiate the target plants. Furthermore, all twelve analytes contribute to the activity of the extracts, including their ability to quench free radicals, reduce metal ions, enhance the non-enzymatic antioxidant capacity of human plasma exposed to oxidative/ nitrative stress, and inhibit oxidative changes in biomolecules (lipids and proteins). The total contribution of the set is in part the result of additive antioxidant effects and in part an effect of synergy between the individual constituents. At the concentration ratios similar to those observed in the extracts, the best synergy exists between ECA and CHA as representatives of flavanols and caffeic acid derivatives, with some effects also visible for the mixtures of QPH as the model flavonol. The levels of all three fractions should therefore be standardized in the *Cotoneaster* extracts in the case of their therapeutic application. The proposed HPLC-PDA method, fully validated during the present study, using twelve active markers as calibration standards, might be recommended as a fast, simple, accurate and reproducible tool, suitable for that purpose. As the method uses gradient elution, a series of system suitability parameters, such as resolution factors (*Rs*), symmetry factors (*T*) and *RSD* values for retention times of the markers should be regularly monitored to ensure that the validity of the analytical procedure is maintained whenever applied. In addition, considering the significant contribution of the high-molecular-weight flavanols to the composition and activity of the tested extracts, the level of these compounds should also be controlled with the proper methodology.

## Figures and Tables

**Table 1:** Linearity, sensitivity, precision and accuracy data for the proposed HPLC-PDA method.

Analyte	t_R_	λ (nm)	Linear Range (µg/mL)	Regression			LOD (µg/mL)	LOQ (µg/mL)	Precision		Recovery (%)
				Calibration Equation	*n*	*r*			Intra-Day (RSD, %)	Inter-Day (RSD, %)	
**NCHA**	3.58	325	1.85–110.5	*y* = 14,045.0*x*	6	0.9997	0.402	1.217	1.25	1.72	97.0
**CHA**	6.19	325	2.65–265.30	*y* = 14,195.0*x*	7	0.9997	0.397	1.204	1.40	1.75	100.5
**CCHA**	6.82	325	1.55–105.4	*y* = 13,934.0*x*	6	0.9998	0.405	1.227	1.30	1.65	98.4
**PB2**	7.33	280	1.65–82.30	*y* = 2399.4*x*	6	0.9998	0.144	0.436	2.67	3.31	97.4
**ECA**	7.91	280	1.49–149.40	*y* = 3367.1*x*	7	0.9999	0.077	0.233	2.02	2.63	99.2
**PC1**	8.71	280	1.52–78.30	*y* = 2299.8*x*	6	0.9997	0.135	0.403	2.70	3.51	98.6
**CAD**	9.12	328	1.92–95.80	*y* = 5251.7*x*	6	0.9997	0.071	0.220	1.35	1.69	97.5
**QPH**	9.54	350	2.16–08.20	*y* = 6398.0*x*	6	0.9998	0.018	0.054	1.83	2.01	98.6
**RT**	10.23	350	1.15–114.90	*y* = 7135.3*x*	7	0.9998	0.062	0.187	1.30	1.61	99.3
**HP**	10.49	350	2.51–251.20	*y* = 9942.0*x*	7	0.9998	0.129	0.391	1.25	1.63	98.2
**IQ**	10.85	350	1.39–69.60	*y* = 9786.2*x*	6	0.9997	0.047	0.142	1.78	1.94	96.9
**QR**	11.83	350	1.01–50.60	*y* = 8929.3*x*	6	0.9997	0.006	0.017	1.93	2.16	97.1

Codification of the phenolic compounds is given in Figure 1. λ, the wavelength at which quantitative determinations were performed; Parameters of the regression model: *y* = a*x*; *y*, peak area; *x*, concentration of standards in µg/mL; *n*, number of data points (concentration levels); *r*, correlation coefficient; LOD, limit of detection; LOQ, limit of quantitation. Precision data are presented for the lowest analyte concentration tested. Recovery data refer to the spiked levels of the analyte added to the sample of *C. bullatus* leaves.

**Table 2:** The quantitative profile of the methanol-water (7:3, *v*/*v*) extracts of *C. bullatus* and *C. zabelii* leaves.

Analyte	MEB	MEZ	Fraction	MEB	MEZ
	mg/g dw			mg/g dw	
**NCHA**	3.56 ± 0.11 ^C^	1.98 ± 0.02 ^A^	**TCA**	23.46 ± 1.57	47.36 ± 0.54
**CHA**	16.30 ± 0.92 ^H^	9.14 ± 0.13 ^F^	**TFL**	18.95 ± 0.94	14.91 ± 0.05
**CCHA**	3.46 ± 0.18 ^C^	2.67 ± 0.05 ^B^	**TLPA**	53.71 ± 3.78	39.76 ± 0.50
**PB2**	17.60 ± 1.20 ^I^	11.93 ± 0.22 ^G^	**TPH**	96.12 ± 5.19	102.03 ± 1.09
**ECA**	12.07 ± 0.78 ^F^	8.89 ± 0.13 ^E^	**TPA (CYE)**	239.62 ± 12.36	241.84 ± 7.27
**PC1**	14.28 ± 0.85 ^G^	8.88 ± 0.10 ^E^			
**CAD**	-	31.83 ± 0.32 ^H^			
**QPH**	7.50 ± 0.36 ^E^	-			
**RT**	0.26 ± 0.01 ^A^	3.36 ± 0.02 ^C^			
**HP**	5.66 ± 0.28 ^D^	1.72 ± 0.02 ^A^			
**IQ**	0.80 ± 0.04 ^B^	2.36 ± 0.02 ^B^			
**QR**	3.30 ± 0.17 ^C^	4.34 ± 0.04 ^D^			

Data expressed as mean values ± SD (*n* = 3). For each analyte, different superscript capitals (^A–H^) indicate significant differences (*p* < 0.05). Codification of the compounds is given in Figure 1. Abbreviations: MEB, MEZ: methanol-water (7:3, *v*/*v*) extracts of *C. bullatus* and *C. zabelii* leaves, respectively; TCA, total content of caffeic acid derivatives; TFL, total flavonoid content; TLPA, total content of low-molecular flavanols and proanthocyanidins; TPH, total phenolic content (HPLC-PDA peaks); TPA, total content of proanthocyanidins (*n*-butanol/HCl assay) expressed in CYE and adopted from [6].

**Table 3:** Antioxidant activity of *Cotoneaster* polyphenols in chemical models.

Analyte	Radical Scavenging Activity DPPH ^a^			Reducing Power ^b^			LA-Peroxidation TBARS ^c^		
	SC_50_		TE	FRAP		TE	IC_50_		TE
	µg/mL	µmol/L	mmol TE/g	mmol Fe^2+^/g	mol/mol	mmol TE/g	µg/mL	µmol/L	mmol TE/g
**CHA**	4.60 ± 0.07 ^G^	12.97 ± 0.21 ^F^	3.51 ± 0.05 ^B,C^	25.68 ± 0.49 ^E^	9.10 ± 0.18 ^E^	9.75 ± 0.26 ^G^	2.49 ± 0.01 ^A,B^	6.83 ± 0.08 ^D^	13.65 ± 0.19 ^G,H^
**PB2**	2.46 ± 0.03 ^B^	4.25 ± 0.05 ^B^	6.57 ± 0.08 ^G^	32.64 ± 0.80 ^F^	18.89 ± 0.47 ^H^	12.63 ± 0.37 ^H^	2.56 ± 0.18 ^A,B^	4.43 ± 0.32 ^B^	13.28 ± 0.95 ^G,H^
**ECA**	2.35 ± 0.10 ^B^	8.08 ± 0.50 ^E^	6.89 ± 0.41 ^G,H^	35.79 ± 0.93 ^G^	10.39 ± 0.27 ^F^	13.97 ± 0.43 ^H^	2.25 ± 0.19 ^A^	7.73 ± 0.66 ^E^	15.16 ± 1.29 ^H^
**PC1**	2.55 ± 0.11 ^B^	2.94 ± 0.09 ^A^	6.34 ± 0.28 ^G^	21.08 ± 0.80 ^C^	18.27 ± 0.06 ^H^	7.35 ± 0.37 ^D,E^	2.91 ± 0.08 ^A,B^	3.35 ± 0.09 ^A^	11.68 ± 0.31 ^F,G^
**CAD**	3.76 ± 0.11 ^E^	12.47 ± 0.37 ^F^	4.38 ± 0.13 ^D,E^	22.31 ± 1.22 ^C,D^	6.61 ± 0.36 ^C^	7.76 ± 0.56 ^E,F^	3.53 ± 0.28 ^B,C^	11.90 ± 0.93 ^G^	9.65 ± 0.76 ^E,F^
**QPH**	5.09 ± -0.08 ^H^	8.54 ± 0.14 ^E^	3.18 ± 0.05 ^B^	15.71 ± 0.20 ^B^	9.37 ± 0.12 ^E^	5.98 ± 0.13 ^C,D^	5.00 ± 0.24 ^D,E,F^	8.39 ± 0.40 ^E^	6.79 ± 0.33 ^C,D^
**RT**	4.44 ± 0.05 ^G^	7.27 ± 0.08 ^D^	3.64 ± 0.04 ^B,C^	15.68 ± 0.62 ^B^	9.57 ± 0.38 ^E^	5.79 ± 0.32 ^C^	6.01 ± 0.46 ^F^	9.84 ± 0.75 ^F^	5.66 ± 0.43 ^B,C^
**HP**	3.42 ± 0.07 ^C,D,E^	7.83 ± 0.16 ^D^	4.73 ± 0.10 ^E,F^	20.64 ± 0.02 ^C^	9.01 ± 0.01 ^E^	7.87 ± 0.08 ^E,F^	5.43 ± 0.62 ^E,F^	12.55 ± 1.28 ^G^	6.29 ± 0.72 ^C,D^
**IQ**	3.55 ± 0.08 ^D,E^	8.12 ± 0.21 ^E^	4.56 ± 0.10 ^D,E^	19.69 ± 0.27 ^C^	8.59 ± 0.06 ^D^	7.37 ± 0.11 ^D,E^	5.89 ± 0.09 ^F^	13.50 ± 0.36 ^H^	5.76 ± 0.09 ^B,C^
**QR**	3.31 ± 0.03 ^C,D^	7.39 ± 0.08 ^D^	4.88 ± 0.05 ^E,F^	20.26 ± 0.36 ^C^	9.08 ± 0.16 ^E^	7.78 ± 0.17 ^E,F^	4.15 ± 0.10 ^C,D^	9.04 ± 0.52 ^F^	8.18 ± 0.20 ^D,E^
**QU**	1.85 ± 0.11 ^A^	6.31 ± 0.33 ^C^	8.74 ± 0.07 ^H^	46.24 ± 1.66 ^H^	13.98 ± 0.50 ^G^	18.10 ± 0.99 ^I^	1.76 ± 0.06 ^A^	5.89 ± 0.29 ^C^	19.28 ± 0.62 ^I^
**TX**	4.06 ± 0.11^F^	16.20 ± 0.42 ^G^	3.99 ± 0.10 ^C,D^	12.69 ± 0.42 ^B^	3.18 ± 0.11 ^A^	4.31 ± 0.19 ^B^	8.51 ± 0.74 ^G^	33.66 ± 2.48 ^J^	4.00 ± 0.35 ^A,B^
**AA**	3.13 ± 0.05 ^C^	17.63 ± 0.04 ^H^	5.17 ± 0.08 ^F^	25.26 ± 0.15 ^D,E^	4.45 ± 0.01 ^B^	9.19 ± 0.01 ^F,G^	4.59 ± 0.27 ^C,D,E^	26.06 ± 0.80 ^I^	7.40 ± 0.43 ^C,D,E^
**MEB**	7.19 ± 0.32 ^I^	-	3.06 ± 0.14 ^A^	10.74 ± 0.07 ^A^	-	3.76 ± 0.03 ^A^	10.58 ± 0.32 ^H^	-	3.21 ± 0.10 ^A^
**MEZ**	7.44 ± 0.03 ^I^	-	2.96 ± 0.01 ^A^	9.42 ± 013 ^A^	-	3.15 ± 0.06 ^A^	11.43 ± 0.71 ^H^	-	2.97 ± 0.18 ^A^

Results are expressed as the means ± SD (*n* = 3). In each column, different superscript letters (^A–J^) indicate significant differences in the mean values (*p* < 0.05). Codification of the compounds is given in Figure 1 and Table 2. ^a^ Radical-scavenging efficiency expressed as effective concentration (SC_50_, amount of the analyte needed to decrease the initial DPPH concentration by 50%). ^b^ Ferric reducing antioxidant power. ^c^ Ability to inhibit linoleic acid peroxidation, monitored by the levels of thiobarbituric acid-reactive substances (TBARS) and expressed as IC_50_ (inhibitory concentration). TE, Trolox^®^ equivalent antioxidant activity. Positive controls: QU, quercetin; TX, Trolox^®^; AA, ascorbic acid.

**Table 4:** Contribution of individual phenolics and groups of phenolics to the antioxidant activity (FRAP) of the *C. bullatus* and *C. zabelii* extracts in chemical and biological models.

Analyte	Ferric Reducing Antioxidant Power (FRAP)					
	Chemical Model			Human Plasma Model		
	AE ^a^ (g/g)	CT_CB_^b^ (%)	CT_CZ_^b^ (%)	AE ^a^ (g/g)	CT_CB_^b^ (%)	CT_CZ_^b^ (%)
**NCHA**	1.02	0.85	0.54	1.45	0.82	0.44
**CHA**	1.02	3.90	2.49	1.45	3.77	2.03
**CCHA**	1.02	0.83	0.73	1.45	0.80	0.59
**PB2**	1.29	5.35	4.12	1.47	4.12	2.68
**ECA**	1.42	4.03	3.38	1.57	3.01	2.13
**PC1**	0.83	2.81	1.99	1.63	3.69	2.21
**CAD**	0.88	-	7.53	1.04	-	5.04
**QPH**	0.62	1.10	-	0.60	0.71	-
**RT**	0.62	0.04	0.57	0.51	0.02	0.27
**HP**	0.82	1.10	0.37	0.80	0.73	0.21
**IQ**	0.78	0.17	0.50	0.32	0.04	0.12
**QR**	0.80	0.62	0.92	0.58	0.30	0.38
**TCA**	-	5.61	11.77	-	5.42	8.49
**TFL**	-	3.30	3.05	-	1.99	1.36
**TLPA**	-	14.10	11.75	-	13.34	9.53
**TPH**	-	23.01	26.57	-	20.75	19.38
**TPA**	-	47.03	54.12	-	61.89	60.17
**MEB**	0.38	100	-	0.63	100	-
**MEZ**	0.32	-	100	0.65	-	100

^a^ FRAP values for a given analyte from the chemical (Table 3) and biological (Figure 4, concentration level: 50 µg/mL) model, expressed in ascorbic acid equivalents (AE). ^b^ Percentage contribution of individual phenolics and group of phenolics to the activity of the extracts from *C. bullatus* (*CT*_CB_) and *C. zabelii* (*CT*_CZ_), calculated according to Equation (1) assuming pure additive interactions. For compounds, phenolic fractions and extracts codes, see Figure 1 and Table 2.

**Table 5:** Interactions between the representatives of the dominant polyphenols of *C. bullatus* and *C. zabelii* leaves.

Phenolic Mixture	Concentration Ratio (µg/mL)	Theoretical Effective Concentration (µg/mL)	Experimental Effective Concentration (µg/mL)	IF ± 95% Conf.	Effect
**ECA:QPH**	1:4	2.09	1.81 ± 0.01	0.93 ± 0.01	synergy
	1:2	1.80	1.81 ± 0.07	1.04 ± 0.04	additivity
	1:1	1.74	1.52 ± 0.01	0.98 ± 0.01	additivity
	2:1	1.48	1.38 ± 0.01	0.99 ± 0.01	additivity
	4:1	1.39	1.17 ± 0.01	0.91 ± 0.01	synergy
**ECA:CHA**	1:4	1.56	1.36 ± 0.03	0.89 ± 0.02	synergy
	1:2	1.39	1.23 ± 0.01	0.85 ± 0.01	synergy
	1:1	1.41	1.24 ± 0.01	0.91 ± 0.01	synergy
	2:1	1.25	1.15 ± 0.08	0.89 ± 0.06	synergy
	4:1	1.26	1.04 ± 0.13	0.84 ± 0.10	synergy
**CHA:QPH**	1:4	2.19	2.00 ± 0.02	0.93 ± 0.01	synergy
	1:2	1.97	1.89 ± 0.02	0.92 ± 0.01	synergy
	1:1	1.99	1.99 ± 0.09	1.03 ± 0.05	additivity
	2:1	1.78	1.87 ± 0.09	1.02 ± 0.05	additivity
	4:1	1.79	1.81 ± 0.19	1.03 ± 0.11	additivity

Results are mean values ± SD (*n* = 3). *IF*, interaction factor, calculated according to Equation (2). Codification of the phenolic compounds is given in Figure 1.

## Supplementary Materials

The following are available online at https://www.mdpi.com/2076-3921/9/1/69/s1, Figure S1: Representative UHPLC-UV chromatograms of the methanol-water (7;3, *v*/*v*) extracts from the leaves of *C. bullatus* **(a)** and *C. zabelii* **(b)** at 280 nm. Table S1: UHPLC-PDA-ESI-MS^3^ data of polyphenols identified in the methanol-water (7:3, *v*/*v*) extracts from the leaves of *C. bullatus* and *C. zabelii*. Table S2: Chromatographic properties of the optimized HPLC-PDA method.

## Abbreviations

UHPLC: Ultra-high performance liquid chromatography; HPLC: High-performance liquid chromatography; PDA: Photo-diode array; ESI: Electrospray ionization; MS: Mass spectrometry; NMR: Nuclear magnetic resonance; COSY: Correlated spectroscopy; HMBC: Heteronuclear multiple bond correlation; HMQC: Heteronuclear multiple quantum correlation; TLC: Thin-layer chromatography; DPPH: 2,2-diphenyl-1-picrylhydrazyl; FRAP: Ferric reducing antioxidant power; TBARS: Thiobarbituric acid-reactive substances; 3-NT: 3-Nitrotyrosine; -SH groups: Free thiol groups; NEAC: Nonenzymatic antioxidant capacity of plasma.
