# Supplementary material for: Contribution of Individual Polyphenols to Antioxidant Activity of Cotoneaster bullatus and Cotoneaster zabelii Leaves—Structural Relationships, Synergy Effects and Application for Quality Control
Source: Antioxidants (Basel). 2020 Jan 12;9(1):69. doi: 10.3390/antiox9010069 (PMC7023040; doi:10.3390/antiox9010069)
Supplement: Supplementary file 1 [file antioxidants-09-00069-s001.pdf]

antioxidants

Supplementary Materials:

Contribution of Individual Polyphenols to Antioxidant Activity of *Cotoneaster bullatus* and *Cotoneaster zabelii* Leaves - Structural Relationships, Synergy Effects and Application for Quality Control

Agnieszka Kicel, Aleksandra Owczarek, Paulina Kapusta, Joanna Kolodziejczyk-Czepas, and Monika A. Olszewska

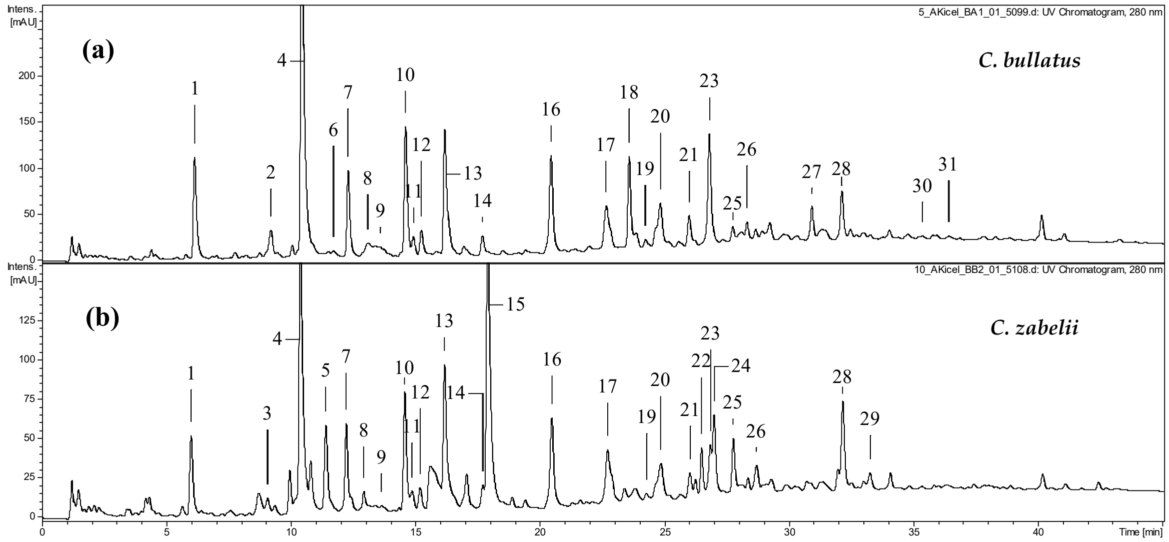

Figure S1. Representative UHPLC-UV chromatograms of the methanol-water (7;3, *v/v*) extracts from the leaves of *C. bullatus* **(a)** and *C. zabelii* **(b)** at 280 nm. The peak numbers refer to those applied in Table S1.

Table S1. UHPLC-PDA-ESI-MS^3 data of polyphenols identified in the methanol-water (7:3, *v*/*v*) extracts from the leaves of *C. bullatus* and *C. zabelii.*

Peak	Analyte	R_t [a] (min)	UV λ_{max} [b] (nm)	$[M-H]^-$ [c] (m/z)	MS^2	MS^3	ME Extracts [d]
**1**	neochlorogenic acid (3-*O*-caffeoylquinic acid, **NCHA**) *	6.1	325	353	191, 179, 135		CB, CZ
**2**	procyanidin dimer B-type	9.0	280	577	451, **425**, 407, 289	407, 273	CB
**3**	caffeic acid derivative	9.7	325	451	405, 179		CZ
**4**	chlorogenic acid (5-*O*-caffeoylquinic acid, **CHA**)*	10.4	325	353	191, 179		CB, CZ
**5**	dicaffeoylquinic acid isomer	11.1	325	515	395, 379, 285		CZ
**6**	procyanidin dimer B-type	11.7	280	577	451, **425**, 407, 289	407, 273	CB
**7**	cryptochlorogenic acid 4-*O*-caffeoylquinic acid, **CCHA**)*	12.3	325	353	191, 179, 173		CB, CZ
**8**	dicaffeoylquinic acid isomer	12.8	325	515	395, 379, 285		CB, CZ
**9**	procyanidin dimer B-type	13.5	280	577	451, **425**, 407, 289	407, 273	CB, CZ
**10**	procyanidin B2 (**PB2**)*	14.6	280	577	451, **425**, 407, 289	407, 273	CB, CZ
**11**	procyanidin trimer B-type	15.0	280	865	739, **713**, 695, 577	695, 425, 407	CB, CZ
**12**	5-*p*-coumaroylquinic acid	15.3	310	337	191, 163		CB, CZ
**13**	(-)-epicatechin (**ECA**)*	16.1	280	289	245, 205, 179, 137		CB, CZ
**14**	procyanidin tetramer B-type	17.7	280	1153	1027, 863, 739, 501, 491, 289		CB, CZ
**15**	caffeic acid derivative (**CAD**)	18.2	290, 328	613	457, 339, 295, 179		CZ
**16**	procyanidin C1 (**PC1**)*	20.4	280	865	**713**, 695, 577	695, 425, 407	CB, CZ
**17**	procyanidin tetramer B-type	22.9	280	1153	1027, 863, 739, 501, 491, 289		CB, CZ
**18**	quercetin pentoside-hexoside (**QPH**)	23.6	268, 355	595	463, 445, 301		CB
**19**	procyanidin tetramer B-type	24.3	280	1153	1027, 863, 739, 501, 491, 289		CB, CZ
**20**	procyanidin dimer hexoside	24.9	280	739	587, 577, 451, 289		CB, CZ
**21**	quercetin rhamnoside-hexoside	26.4	255, 355	609	**447**, 343, 301	301	CB, CZ
**22**	quercetin dirhamnoside	26.6	255, 350	593	**447**, 301	301	CZ
**23**	hyperoside (quercetin 3-*O*-ß-galactoside, **HP**)*	26.9	255, 353	463	301		CB, CZ
**24**	rutin (quercetin 3-*O*-ß-(6′′-*O*-α-rhamnosyl)-glucoside , **RT**)*	27.3	265, 350	609	463, 343, 301		CZ
**25**	isoquercitrin (quercetin-*O*-ß-glucoside, **IQ**)*	27.9	275, 350	463	301		CB, CZ
**26**	procyanidin dimer B-type	28.5	280	577	425, 407, 289		CB, CZ
**27**	quercetin rhamnoside-hexoside	31.0	265, 355	609	447, 301		CB
**28**	quercitrin (quercetin 3-*O*-ß-rhamnoside, **QR**)*	32.2	275, 350	447	301		CB, CZ
**29**	quercetin hexoside derivative	33.3	265, 355	505	463, 337, 301		CZ
**30**	quercetin dirhamnoside	35.3	365, 355	593	447, 301		CB
**31**	dicaffeoylquinic acid isomer	36.3	325	515	379, **353**, 299, 203	191, 179, 173	CB

* identified with authentic standards; [a] R_t, retention time; [b] UV λmax, absorbance maxima in PDA spectra; [c] $[M-H]^-$, pseudomolecular ion in MS spectra recorded in a negative mode; [d] **ME** extracts, methanol-water (7:3, *v*/*v*) leaf extracts; **CB**, *C. bullatus*; **CZ**, *C. zabelii.*

Table S2. Chromatographic properties of the optimized HPLC-PDA method.

Analyte	t_R (*min*)	*RSD* t_R (%)	R_s	*T*	*w*	*F*
**NCHA**	3.58	2.28	-	1.021	2.616	1.281
**CHA**	6.19	0.88	16.337	1.038	1.558	0.750
**CCHA**	6.82	0.58	5.242	1.052	1.460	0.694
**PB2**	7.33	0.99	5.125	1.036	1.409	0.680
**ECA**	7.91	0.48	5.785	1.143	1.215	0.532
**PC1**	8.71	1.04	7.601	1.061	1.361	0.641
**CAD**	9.12	1.54	3.102	1.121	1.260	0.562
**QPH**	9.54	1.12	6.914	1.308	1.260	0.482
**RT**	10.23	0.53	8.787	1.087	1.111	0.511
**HP**	10.49	0.66	1.703	1.112	1.158	0.520
**IQ**	10.85	0.70	2.022	1.109	1.142	0.515
**QR**	11.83	0.82	12.006	1.119	1.215	0.543

t_R, retention time; *RSD* t_R values for retention times (t_R); resolution (R_s) and symmetry (*T*) factors between analyte peaks were calculated using the following equations: $R_s = 2.0 \times (t_{R2} - t_{R1})/(w_2 + w_1)$ and $T = w/2F$, where $(t_{R2} - t_{R1})$ is the difference between retention times of two peaks; $(w_2 + w_1)$ is the sum of peak widths at baseline between tangent lines drawn at 50% of peak heights; *w* is the peak width at 5% of peak height; and *F* is the time from width start point at 5% of peak height to t_R.
